# The Role of Quorum Sensing Molecules in Bacterial–Plant Interactions

**DOI:** 10.3390/metabo13010114

**Published:** 2023-01-10

**Authors:** Jan Majdura, Urszula Jankiewicz, Agnieszka Gałązka, Sławomir Orzechowski

**Affiliations:** Department of Biochemistry and Microbiology, Institute of Biology, Warsaw University of Life Sciences-SGGW, Nowoursynowska 159, 02-787 Warsaw, Poland

**Keywords:** QS signaling molecules, quorum quenching, bacterial–plant interactions

## Abstract

Quorum sensing (QS) is a system of communication of bacterial cells by means of chemical signals called autoinducers, which modulate the behavior of entire populations of Gram-negative and Gram-positive bacteria. Three classes of signaling molecules have been recognized, Al-1, Al-2, Al-3, whose functions are slightly different. However, the phenomenon of quorum sensing is not only concerned with the interactions between bacteria, but the whole spectrum of interspecies interactions. A growing number of research results confirm the important role of QS molecules in the growth stimulation and defense responses in plants. Although many of the details concerning the signaling metabolites of the rhizosphere microflora and plant host are still unknown, Al-1 compounds should be considered as important components of bacterial–plant interactions, leading to the stimulation of plant growth and the biological control of phytopathogens. The use of class 1 autoinducers in plants to induce beneficial activity may be a practical solution to improve plant productivity under field conditions. In addition, researchers are also interested in tools that offer the possibility of regulating the activity of autoinducers by means of degrading enzymes or specific inhibitors (QSI). Current knowledge of QS and QSI provides an excellent foundation for the application of research to biopreparations in agriculture, containing a consortia of AHL-producing bacteria and QS inhibitors and limiting the growth of phytopathogenic organisms.

## 1. Introduction

Quorum sensing (QS) is a bacterial mechanism responsible for cell communication through chemical signals. This phenomenon occurs when there is a sufficiently high density of cells in a specific bacterial habitat. It was discovered and described about half a century ago in the Gram-negative bacterium *Vibrio fisheri* [1,2].

It has now been proven that this chemical communication system occurs in both Gram-negative and Gram-positive bacteria. This phenomenon involves the production and transport of signaling molecules, known as autoinducers (AI), into the intercellular space. AI molecules, depending on their structure, can be transported from the cytoplasm to the outside of the cell by diffusion or active transport. When the concentration of signaling molecules exceeds a threshold, changes in gene expression and metabolic effects occur throughout the bacterial population. QS molecules are responsible for both interspecies communication and interaction with higher organisms; they also regulate cellular processes, such as replication of bacterial DNA, energy metabolism, synthesis of enzymes, polysaccharides and antibiotics’ conjugative transfer of plasmids, bioluminescence, and the motility of microorganisms [3,4,5]. Data from the literature demonstrate that signaling molecules can significantly affect the expression of genes essential for bacterial life and growth. These are genes responsible for, among other things, adaptations to a particular environment, bacterial biofilm organization, horizontal gene transfer, synthesis of toxins and virulence factors, and other compounds involved in interactions between different species [4,6,7,8]. In addition, the type and quality of the bacterial habitat are associated with the concentration of signaling molecules, and consequently with the expression of genes responsible for bacterial adaptation to environmental conditions. This makes autoinducers a necessary and essential tool for evolution to effectively control gene expression, and thus, the behavior of the entire bacterial population [9]. The bacterial autoinducers used in communication have different molecular structures, which determines their classification into classes. These differences exist not only between Gram-positive and Gram-negative bacteria, but also between different bacterial species [10]. Previously, Gram-positive bacteria were thought to use peptides as autoinducers and Gram-negative bacteria used N-acyl-L-homoserine (AHL)-lactones. However, studies in recent years have shown that both groups of bacteria have the ability to synthesize AHL [11,12,13,14].

It is this type of signaling molecule—AHL—that plays an important role in bacterial–plant interactions. Both Gram-negative and Gram-positive bacteria use autoinducer-2 (AI-2) for interspecific communication, and the derivative of 4,5-dihydroxy-2,3-pentodione [15]. The presence of these autoinducers in the rhizosphere zone of plants may induce growth-promoting effects and the plant’s resistance to phytopathogens [16,17,18]. Increasing knowledge of the role of QS molecules in bacterial–plant interactions is driven by the need for applications. Degradative enzymes or inhibitors appear to be extremely useful in regulating this specific communication. Therefore, information on QS molecules and their effects on plant growth was compiled in this work. Information has also been compiled on how QS exerts control by quenching chemical signals.

## 2. Types and Importance of Signaling Molecules

**Figure 1:**
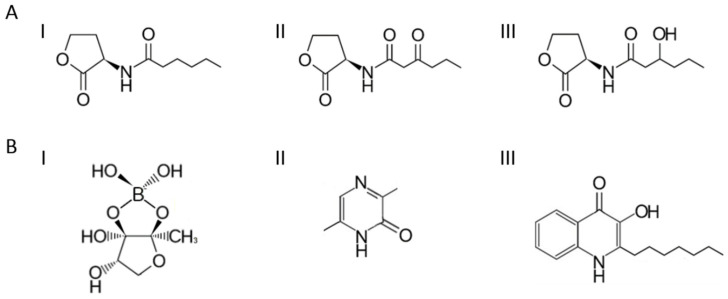
Structure of the different types of autoinducers: (**A**) (**I**) Al-1 unsubstituted homoserine lactone (C6 HSL); (**II**) Al-1 oxidized homoserine lactone (3-oxo-C6-HSL); (**III**) hydroxylated homoserine lactone (3-hydroxy-C6-HSL). (**B**) (**I**) Al-2 (furanosyl borate diester); (**II**) Al-3 (Phevalin, a member of the class of pyrazinones); (**III**) other unclassified autoinducers (quinolone derivative, PQS).

The previously known QS signaling molecules have been divided into three classes: AI-1, AI-2 and AI-3. The autoinducers from each group differ in their chemical structure and functions [10,19]. According to this classification, the AI-1 group is responsible for interspecies communication, AI-2 for interspecies communication, and the AI-3 group for interactions with higher organisms. AI-1 autoinducers are categorized in terms of their molecular structure as N-acyl-L-homoserine lactones (AHLs). Such a molecule is composed of a homoserine lactone (HSL) with a fatty acid placed in the α position. The variation in structure in this group of structures is due to the different number of carbon atoms from 4 to 18 but also to the degree of oxidation, hydroxylation, and the number of unsaturated bonds (Figure 1A). Natural AHLs with fatty acid chain lengths of six to eight carbon atoms are the most abundant [20,21,22,23]. The shortest signaling molecules, consisting of four carbon atoms in the chain, were detected in *Pseudomonas aeruginosa* (N-butyryl-L-homoserine lactone (C4-HSL) and *Vibrio harveyi* (N-3-hydroxy-butyryl-L-homoserine lactone (3-hydroxy-C4-HSL) [24,25]. Long AHL chains with 14 to 18 carbon atoms are characterized by one or two double bonds and are synthesized by many bacteria e.g., *Rhodobacter capsulatus*, *Escherichia coli*, and *Enterobacter cloacae* [26,27,28,29]. Data from the literature suggest a relationship between the length of the fatty acid carbon chain and the properties of the AI as well as its transport outside the cell. Short AHLs with up to six carbon atoms can be transported out of the cell by diffusion, whereas AHLs with more than six carbon atoms in the structure require transporters, such as proton pumps, to ensure their active transport out of the cell through the cell membrane [30,31]. The stability of AHL and its derivatives is highly dependent on the pH of the surrounding environment. These molecules are stable at a pH of 7.0, while an increasing pH causes their hydrolysis [32,33,34].

The autoinducers of the second group were detected in Gram-negative and Gram-positive bacteria. Al-2 has been found to exacerbate the symptoms of acute pneumonia, caused by *P. aeruginosa* [35]. Al-2 is transported out of the cell into the environment by active transport due to the size of the molecules [36]. A well-studied AI-2 is the furanosyl borate diester discovered in *V. harveyi* (Figure 1(BI)) [37].

The autoinducer AI-3, a pyrazinone derivative, was isolated from the culture of *Escherichia coli* (EHEC) serotype O157: H7 (Figure 1(BII)) [38].The presence of such molecules was also observed in cultures of *Shigella* sp. and *Salmonella* sp., as well as in normal intestinal bacterial flora. They enable commensal bacteria to communicate with host cells and with bacteria of another species [39,40].

In addition to these three well-known types of molecules in bacterial communication, autoinducers of a different structure, often characteristic of specific species of bacteria, were also identified. These are, i.e., 3OH palmitic acid methyl ester (3-OH PAME), cyclic dipeptides, quinolone signal molecule in *Pseudomonas* (Figure 1(BIII), PQS), diffusible signal factor (DSF), and cholerae autoinducer-1 (CAI-1) [15,41,42]. Indole is an interesting example of an autoinducer involved in bacterial–host interactions [43].

## 3. Mechanism of the Synthesis and Action of Autoinducers

The pathways of autoinducer biosynthesis, especially Al-1, have been well characterized in many bacterial strains [44,45]. Enzymes from three families, LuxI, HdtS and LuxM, are involved in this process. The best-characterized LuxI is highly conserved, which may indicate limitations in the diversity of AHL-type molecules. In contrast, the LuxR protein, a transcription regulator, has a highly variable sequence during *Luxl* synthesis [22,46].

**Figure 2:**
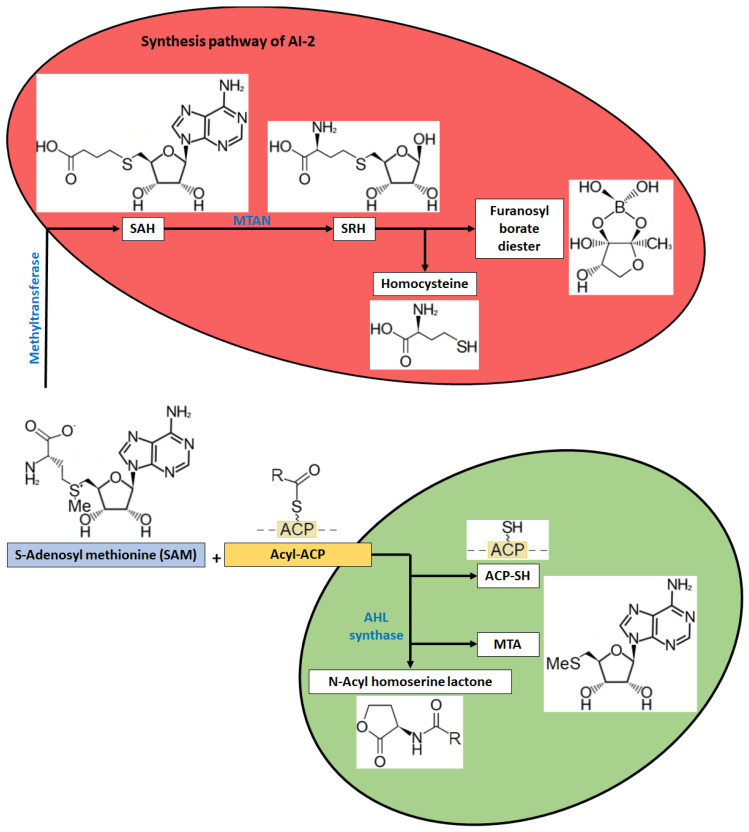
Schematic of the biosynthetic pathway of Al-1 and Al-2 signaling molecules. SAM-S-adenosyl-L-methionine, MTA-5’-methylthioadenosine nucleosidase, MTAN-5’-methylthioadenosine nucleosidase, SAH-S-adenosylhomocysteine, SRH-S-ribosylhomoserine (SRH).

AHL synthases, LuxI catalyze the reaction of AHL synthesis using substrates such as S-adenosyl-L-methionine (SAM) and a fatty acid carried by the carrier protein ACP and, in addition, can use acyl-coenzyme A as substrates. Enzymes of the HdtS family are involved in the synthesis of unsubstituted AHL molecules and more complex AIs such as N-(3-OH-7-cis-tetradecenoyl)-HSL [15,20,47]. The first step of the AI-1 synthesis pathway is the binding of SAM to the active center of AHL synthase. This is followed by the transfer of the acyl group from acyl-ACP to the SAM–AHL synthase complex and the formation of an amide bond with the amino group of SAM. The next step is the formation of an ester bond in the homoserine moiety and the formation of two lactone products N-acyl-L-homoserine and 5’-methylthioadenosine (MTA). MTA is a byproduct of this reaction, but can be used by many bacteria, with the involvement of the enzyme 5’-methylthioadenosine nucleosidase (MTAN), to produce adenine and 5-methylthioribose phosphate (MTR-1-P), which is used by bacteria to synthesize SAM [48]. The synthesis of these signaling molecules involves AI-2, which synthesizes S-adenosylhomocysteine (SAH) from SAM. SAH is then converted to S-ribosylhomoserine (SRH) by the enzyme MTAN. This compound is the precursor of tetrahydrofuran in AI-2 molecules (Figure 2) [49,50].

In recent years, a mechanism for the biosynthesis of the Al-3 signaling molecule has been proposed. It is known that the substrate-amino acid derivatives—for the biosynthesis of this pyrazinone molecule—are provided by threonine dehydrogenase and tRNA synthetases. An important role for aminoacyl-AMP (abortive tRNA synthetase products), after spontaneous reaction with aminoketones, in modulating Al-3 transcription has also been demonstrated [38].

### Mechanism of Action of AI-1 Autoinducers in Gram-Negative Bacteria

The molecular mechanisms of the QS phenomenon often depend on the species and the environment in which they occur. The bacterium V. fischeri has two QS systems that use AHL as signaling molecules: lux (LuxR/LuxI) and ain (AinS/AinR). AI-1 molecules are synthesized by LuxI or LuxM enzymes, and AI-2 molecules are synthesized by LuxS enzyme. LuxI synthase participates in the formation of 3-oxo-C6-HSL, which binds to the activator protein LuxR. The 3-oxo-C6-HSL-LuxR complex, thus formed interacts with the promoter of the luxICDABEG operon and induces the expression of these genes [46,51]. The LuxR/LuxI system is responsible for the expression of luminescence in vivo and the maintenance of the microsymbiont within the host organ. The second QS system of V. fischeri is based on the AinS protein with AHL synthase activity, which belongs to the LuxM family. This enzyme synthesizes N-octanoyl-homoserine lactone (C8-HSL), which is recognized by the transcription factor AinR [52]. At low bacterial cell population densities, the genes responsible for the luminescence phenomenon are repressed by the LuxO protein, which is a negative regulator of the gene encoding the transcriptional regulator LitR. As the bacterial population density increases, the signaling molecule is synthesized by AinS. As the bacterial population density increases, the signaling molecule synthesized by AinS causes two effects: the induction of luminescence gene expression through its direct interaction with LuxR and the inactivation of LuxO protein and an increase in the transcription level of the *litR* gene. LitR positively regulates *luxR* transcription, and thus, functionally links the two lux and ain systems, ensuring the gradual induction of luminescence-related gene expression as the bacterial population density increases under symbiosis conditions. The ain system is essential for the initiation of colonization of the host organism and is responsible for bacterial motility, while the lux system is involved only in the later stages of symbiotic interactions. Due to the action of these two QS systems, V. fischeri is able to establish symbiotic relationships with the squid Euprymna scolopes when the population of these bacteria reaches a threshold level and the expression of genes regulated by these mechanisms follows. Such regulation of colonization factor gene expression is expressed only when it is beneficial to the bacterial cells, avoiding costly metabolic processes while in an aqueous environment [53,54].

The second well-described mechanism of action of the QS system occurs in bacteria of the genus Pseudomonas, particularly *P. aeruginosa*. This bacterial species is an opportunistic pathogen, exhibits high antibiotic resistance, and is a frequent contributor to nosocomial infections. The QS system of *P. aeruginosa* bacteria is responsible for controlling the synthesis of virulence factors of this bacterium, including LasA protease and aprA, LasB and rhamnosyltransferase, lectin pyocyanin, and toxin A [55,56,57,58]. In this bacterial species, there are three major cooperating systems, two of which utilize N-acylhomoserine lactones (AHL) as signaling molecules, referred to as the las system and the rhl system, respectively. The third system, QS, is called the pqs system and is related to the other two systems. Together, these three systems form a complex quorum sensing system and are interdependent. Among the QS mentioned above, the las system is at the top of the QS hierarchy and is required for optimal activation of the rhl and pqs systems QS. The las system includes two proteins, LasI (synthase) and LasR, while the rhl system includes the proteins RhlI (synthase) and RhlR [59]. Another example is the phytopathogenic soil bacterium Agrobacterium tumefaciens, which uses the determinants of the QS system belonging to the LuxR/LuxI class. In this species, the QS system controls the translocation of Ti plasmid, for which the regulatory proteins Tral and TraR are responsible. The LuxI-like protein TraI synthesizes N-acyl homoserine lactone molecules that act as diffusible QS signals. When a certain threshold concentration is exceeded, these molecules bind and activate the transcriptional regulator TraR [60]. In contrast, the secretion of exoenzymes responsible for the destruction of cell wall structures in the phytopathogenic bacterium Ervinia carotovora is controlled by a system of proteins corresponding to the LuxI/LuxR system [61,62]. Bacteria of the genus Serratia are equipped with four QS systems of LuxI/LuxR depending on the species: SwrI/SwrR, SmaI/SmaR and SpnI/SpnR and the best studied SprI/SprR, typical of S. proteamaculans [55,63].

## 4. QS System Inhibitors and Degrading Enzymes

The QS system inhibitor (QSI) is a natural or synthetic compound that has the ability to silence QS mechanisms, also known as quorum quenching (QQ). QQ can be achieved in several ways: inhibition of the synthesis of signaling molecules, enzymatic degradation of molecules, blocking of receptors that recognize AHL molecules, inhibition of gene expression, and interception of AIs by antibodies and macromolecules such as cyclodextrins [64,65].

AHL synthesis can be inhibited by introducing analogs of SAM, which is essential for AI production, into the cytoplasm, and using purine nucleotide analogs or homoserine lactone derivatives. Degradation of AHL can occur by chemical (alkaline pH reversible process), enzymatic or metabolic means. In contrast, the use of AHL-antagonistic molecules, which can compete for a binding site in the receptor, can effectively block the interaction between the signal molecule and the receptor [55].

We can divide QS inhibitors into QSIs of natural and synthetic origin. Natural QSIs can be further divided into those derived from marine organisms, plants, bacteria or animals [56,57].

Table 1 shows selected natural and synthetic QS inhibitors together with their mechanism of action and effective concentrations.

Among marine organisms, the most important group of QSI-synthesizing organisms is that of marine cyanobacteria. An example of such a bacterium is Delisea pulchra, which synthesizes halogenated furanones [80]. These are compounds that act as competitive analogs of AHL. This is possible due to their structural similarity to short AIs. Studies have shown that they compete with signaling molecules for a binding site in the receptor, but also induce its degeneration in direct interaction with LuxR protein, leading to disruption in the expression of genes dependent on the mechanism’s QS. The result of halogenated furanones is the inhibition of biofilm production by bacteria. Although they are QSIs capable of inhibiting the QS systems of many bacteria, they show toxicity to host cells at higher concentrations [81,82,83].

**Figure 3:**
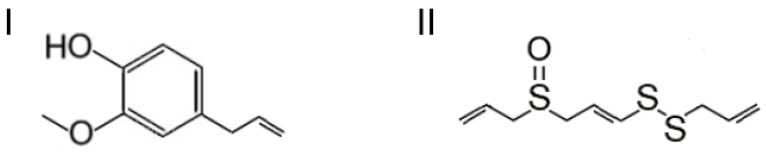
Structure of plant-derived QS inhibitors. (**I**) Eugenol (from cloves and cinnamon); (**II**) Ajoene (from garlic).

In the literature, compounds of plant origin are often considered as one of the most important groups of QSIs. They are characterized by the fact that their chemical structure has many similarities to AHL and they are able to degrade protein transcriptional regulators. Another important factor is their ubiquity; they are found in herbs, vegetables and fruits. In terms of chemical structure, QSIs belong to polyphenols, terpenes, alkaloids, and coumarins [84]. Important producers of QSI are plants from the *Brassicaceae* family, ginger plants, legumes, and medicinal plants. Plants from the *Brassicaceae* family produce sulforaphane, which inhibits the activity of the transcriptional regulator LasR and whose antagonistic activities against AI have been confirmed in *P. aeruginosa* [85]. Inhibitors containing sulfur in their composition are the focus of interest. These are substances derived from garlic, onions, leeks, and cabbage, kale, and broccoli [64,86]. Inhibitors of biofilm formation extracted from plants include eugenol and ajoene (Figure 3) [67,84].

In animals and bacteria, there are three main types of enzymes that can perform QSI functions: AHL lactonases, AHL acylases, and AHL oxidoreductases [64,81,87]. The ability to synthesize the above enzymes has been detected in bacteria from the following groups: Actinobacteria (Rhodococcus, Streptomyces), Firmicutes (Arthrobacter, Oceanobacillus, Bacillus), Bacteroides (Tenacibaculum), Cyanobacteria (Anabaena), Proteobacteria (Comomonas, Acinetobacter, A. tumefaciens, K. pneumoniae, Ralstonia, Alteromonas, Stappia, V. paradaoxus, Halomonas, Hyphomonas), and in animals such as rats, mice and the freshwater fish Danio rerio [64]. AHL lactonases degrade AHL molecules by reversibly hydrolyzing the ester bonds in the lactone ring of homoserine, resulting in products that are acyl-homoserine derivatives (AHS). The next type of AI-degrading enzymes are AHL acylases, which perform irreversible hydrolysis of the amide bond between the L-homoserine lactone and the acyl side chain. This reaction leads to the release of the corresponding fatty acids and homoserine lactone [81]. They are characterized by substrate specificity, usually for long-chain AHLs. The most important difference between the hydrolysis performed by AHL acylases and that performed by AHL lactonases is the formation of a product that cannot be spontaneously converted into a functional AHL molecule [88]. The last type of enzymes with a QSI role are AHL oxidoreductases. Representatives of this group oxidize or reduce functional groups in the acyl side chains of AHL molecules [88,89,90].

An interesting group of QSI producers are soil bacteria, whose ability to degrade QS signaling molecules can effectively prevent the emergence of pathogens and purify the soil of the rhizosphere [91]. In particular, Gram-positive members of the genus *Bacillus* spp. secrete large amounts of AHL lactonases, which can attenuate the virulence of the plant pathogen *Erwinia carotovora* and *Aeromonas hydrophila* YJ-1 [92,93,94]. *Bacillus* spp., and other Gram-positive bacteria that do not produce AHL, show growth inhibition at a high concentration of 3-oxo-C12-homoserine lactone (3-oxo-C12-HSL), so the ecological role of QSI in Gram-positive bacteria is likely to be the detoxification of high concentrations of AHL [95]. There are examples in the literature of the use of AHL-degrading bacteria for biocontrol against plant pathogens. One example is the bacterium *Rhodococcus erythropolis*, whose colonization of potato roots resulted in resistance to *Pectobacterium* [96].

### 4.1. Use of Genetic Engineering and Protein Engineering Methods to Obtain Stable and Active Quorum Quenching Enzymes

Intensive research into enzymes capable of degrading or modifying acyl-homoserine lactones (AHLs) is stimulated by the requirement to develop an effective antimicrobial mechanism to interrupt the bacterial QS process. Such solutions would be useful for human as well as animal and plant health care. Therefore, new methods to obtain and stabilize the activity of Quorum Quenching Enzymes are still under investigation [47,88,97,98]. Recombinant proteins obtained by genetic engineering or improved by protein engineering techniques are increasingly used for such studies. An example of such an enzyme is the recombinant thermostable AHL lactonase AiiM, which was stabilized by electrospinning (ES) an aqueous polyvinyl alcohol (PVA) solution. The test protein was genetically modified by adding a maltose-binding protein (MBP). Immobilization of the AiiM-MBP-lactonase complex resulted in long-term quorum quenching activity against the opportunistic pathogen *S. marcescens* AS-1 [99]. The efficacy of recombinant lactonase in the form of a hydrogel in controlling burn wound infections caused by a multidrug-resistant (MDR) strain of *Pseudomonas aeruginosa* was also demonstrated. These observations raise hopes for a new strategy to eradicate and control wound infections caused by *P. aeruginosa* [100]. Another interesting example is the use of the recombinant lactonase Ai-iAQSI-1 from *Bacillus* sp. to inhibit the virulence of *Aeromonas hyrophila*, an opportunistic pathogen that lives in freshwater and marine environments. The results of this experiment show that the delivery of the exogenous AiiAQSI-1 protein as a component of feed is effective and opens a new avenue in antibacterial therapy [101]. The recombinant lactonase LcAiiK has also shown great efficacy in reducing infections caused by *A. hydrophila* in aquatic cultures [102]. The N-acylhomoserine lactonase-based hybrid AhlX@Ni3(PO4)2 has also been successfully used in the biological control of diseases caused by *Erwinia carotovora* and *Burkholderia glumae* [103].

Protein engineering usually employs the technique of high-throughput screening (HTS), which allows libraries to be created, and thus, enables suitable sequences to be selected. Two approaches are usually used: the use of error prone PCR (epPCR) or rational design approaches [47,104]. The epPCR technique was used to generate a mutant library of the marine-derived quorum quenching (QQ) lactonase. The resulting mutant enzymes showed increased activity in blocking QS signaling in the pathogenic bacterium *Pectobacterium carotovorum*, which causes the soft rot of cabbage [105]. Rational design relies heavily on prior knowledge of the protein’s sequence, structure, and functional data to design specific mutations [106]. This approach has been used to design His6-OPH lactonase with enhanced catalytic performance [107], hyperthermostable lactonase [108], and lactonase with altered substrate specificity [109]. The use of these advanced techniques enables the synthesis of stable and active enzymes, which increases the efficiency in silencing the QS signal.

### 4.2. QSI Applications in Agriculture

AHLs are commonly used by both beneficial and pathogenic rhizobacteria to optimize their beneficial activity or virulence. Therefore, the main plant strategy to avoid or reduce QS-regulated bacterial virulence is to block the receptor using structural analogs of QS signaling molecules categorized as QSIs. For this reason, papers on the use of bacteria to help plants degrade AHL can be found in the literature. Among the *Azospirillum brasilense* strains, which are very often successfully used as plant growth promoting bacteria in practical agriculture in South America [110], the production of AHL autoinducers is very rare [111]. Recently, it was shown that *A. brasilense* Az39 is able to degrade unsubstituted AHLs and AHLs with hydroxyl and ketone groups [112]. It can be speculated that this AHL-degrading activity promotes the competitiveness of Az39 in root colonization and may contribute to the control of plant pathogens dependent on AHL activity. Thus, disruption of QS communication within the rhizosphere community may contribute to the inhibition of pathogen development and the increased competitiveness of plant growth-promoting bacteria such as *A. brasilense* Az39.

## 5. Effects of AHL Compounds on Plant Growth and Health

During the evolution of land plants, plant-associated microbiomes have evolved and been integrated into plant microbial holobionts. The functions of these plant holobionts rely on the expression of genes from all partners. The assembly of the holobiont depends on the emission and perception of signals between the microbes and plants. Presumably, QS molecules are among these signals [113]. The synthesis of AHL molecules released into the environment is subject to self-induction once a threshold is exceeded. This leads to the expression of new cellular phenotypes related to biofilm formation, virulence, symbiosis, and plant interaction. Due to the colonization of plant roots by bacteria, plants have evolved mechanisms to alter their gene expression profile during coevolution, which may subsequently enhance their defense mechanisms against pathogens or lead to cooperation with bacterial saprotrophs [114]. From the literature data, it appears that the effects of AHL-plant interactions vary widely and depend on the structure of the AHL molecule, as summarized in Table 2.

### 5.1. Effects of AHL on Plant Root Morphology

It has been determined that the contact of AHL molecules with a short carbon chain can result in changes in the morphology and phytohormonal balance of roots [115,127]. C10-HSL induced root shortening, and increased the formation of lateral roots and trichomes [121]. In contrast, C6-HSL and C8-HSL increased root length [115,128]. In *A. thaliana*, genes encoding GCR1/GPA1, which are involved in the cell-to-cell transmission of extracellular environmental signals via G proteins, were identified as the genetic basis for the stimulation of root growth by short C6- and C8-HSL. Mutants in GCR1 were insensitive to root growth stimulation by C6- and C8-HSL, while overexpression resulted in enhanced root growth effects [117]. The bacterium *Acidovorax radicis* N35, which produces OH-C10-HSL, is included in the group of plant growth-promoting bacteria. The results obtained in *luxI* deletion mutants suggest that AHL is important in the process of root colonization by these bacteria [129,130]. Significant differences in the interactions with barley plants were also shown. Although the wild type producing AHL induced a change in the expression profile in the plant to stimulating and priming, the AHL deletion mutant resulted in increased expression of defense responses, such as flavonoid biosynthesis [130].

### 5.2. Effects of AHL on the Expression of Plant Genes Associated with Defense Mechanisms

One of the more intrusive types of these interactions is the induction of defense mechanisms in plants in the presence of AHL. In AHL–plant interactions, the expression of certain plant genes is stimulated, resulting in an enhanced defense against pathogens and growth stimulation [127]. In tomatoes inoculated with *Serratia liquefaciens* MG1 and *P. putida* IsoF strains, producing C6- and C8-HSL, defense and biological control activities against the fungal pathogen *Alternaria alternata* have been identified [123]. In plants inoculated with MG1 and wild-type IsoF cells, salicylic acid increased in leaves and induction of SA and ethylene (ET)-dependent defense genes (PR1a and chitinase) was observed. No similar effects were detected after inoculation with *S. liquefaciens* mutants lacking AHL. Moreover, it could be shown that C6- and C8-HSL alone were capable of inducing disease suppression under axenic conditions [123]. Thus, in this system, AHLs are capable of initiating and inducing defense activity in tomato plants. Using fluorescently labeled *S. liquefaciens* MG1 AHLs in combination with AHL reporter bacteria, the production and distribution of AHLs in situ can be monitored in detail [131]. The role of these signaling molecules in the colonization of plant roots by bacteria has also been established. Shrestha and co-workers showed that both 3-oxo-C14-HSL and a combination of other long-chain AHLs induce not only the expression of several plant defense-related genes, but also resistance to the pathogen *P. syringae* [132].

### 5.3. Enhancing Resistance to Pathogens and Insects

Several experimental approaches have shown that water-soluble AHLs are taken up by plants through the plant’s vascular system into the shoot during the process of cellular energy consumption [125,126]. AHL uptake has only been found in plants such as *A. thaliana*, wheat and barley, which are devoid of AHL-degrading enzymes such as lactonases. In plant shoots, hydrophilic AHLs are able to modify the activity of several enzymes, including antioxidant capacity and xenobiotic phase II detoxifying enzymes to improve stress tolerance [126]. Although hydrophobic AHLs (e.g., 3-oxo-C14-HSL) are not absorbed by plants, they confer resistance to the absolute biotrophic fungus *Golovinomyces orontii* in *A. thaliana* and *Blumeria graminis* and to the hemibiotrophic bacterial pathogen *P. syringens pv. tomato* DC3000 [16]. It has been shown that in the presence of the bacterial elicitor flg22, there is an increase in the activity of the protein kinases AtMPK3 and 6, and an increase in the expression of WRKY22 and 29 plus PR1a. Most interestingly, AtMPK6 was required to induce AHL-induced resistance, as deletion mutants did not show an inducible resistance phenotype [16]. The AHL-dependent stimulation of antioxidant gene expression and plant defense activity by hydrophobic AHLs with long carbon chains (3-oxo-C12-HSL or 3-oxo-C14-HSL) occurs through the oxylipin and salicylic acid signaling pathway [128]. Moreover, a role for AHL in pest control was recently demonstrated by inoculating two barley lines with the 3-oxo-C14-HSL-producing bacterium *Ensifer meliloti*. Inoculation of wild-type *E. meliloti* resulted in a reduced feeding and reproductive response of *Rhopalosiphum padiaphids* compared to the AHL-negative mutant and control [133]. This effect of AHL, in turn, reduces the transmission of plant viruses, and thus, contributes to plant health. The first successful field trials with wheat and the application of C6-HSL showed growth stimulation and the potential for practical application to improve crop yield [134]. In addition, C4-HSL applied with carbon nanofibers induced increased growth, stress tolerance and resistance to the fungal pathogen *Fusarium oxysporum* in *Cicer arientinum* [135].

### 5.4. Participation of QS Molecules in the Nitrogen Cycle

Nitrogen is found in such vital molecules as nucleic acids and proteins, making it one of the essential elements for the existence of all life forms. The availability of nitrogen in the soil is a critical factor in plant growth and yield. Both an excess and a deficiency of this element are harmful to plants. Nitrogen is generally supplied to the soil as a component of mineral and organic fertilizers, acid rain, and as a result of biological fixation of atmospheric nitrogen involving nitrogenase produced by some symbiotic and free-living microorganisms. Much of the organic nitrogen in soil consists of macromolecular compounds (chitin, proteins, nucleotides) that are available to plants only after enzymatic depolymerization. The extent of synthesis of these enzymes depends on QS signals and can regulate soil nitrogen cycling and plant nitrogen supply. Diazotrophic bacteria secrete AHLs that are involved in plant–bacteria communication by regulating the rate of the enzymatic hydrolysis of chitin and proteins in soil [136,137]. Nitrogen is a dynamic component that undergoes many transformations in the soil, including its incorporation into organic compounds, ammonification, nitrification, denitrification. The above processes form the nitrogen cycle in nature. In agriculture, there is a tendency to excessive nitrogen fertilization, which leads to water pollution and excessive greenhouse gas emissions. Therefore, the interest of scientists in this field is focused on finding mechanisms to regulate the processes involved in nitrogen transformation in nature. The main microbial processes in the nitrogen cycle, nitrification and denitrification, are regulated by QS-lactone homoserine molecules of different lengths. The authors of many studies emphasize the importance of QS-dependent nitrification and denitrification for the removal of nitrogen from polluted waters [138,139,140,141].

## 6. The Use of Metagenomics to Study QS and QSI (or QQ) Diversity

Advanced research on QS molecules and quorum-quenching molecules is currently focused on the study of metagenomes, especially in soil and marine environments. These are independent of bacterial laboratory cultures. This methodological approach exploits the full genetic potential present in a given microbial habitat and allows the assessment of the abundance and diversity of signaling molecules and their inhibitors. Unfortunately, the results of studies in which compounds are extracted and identified from bacterial cultures are limited by the lack of information on uncultured microbial species, which in some environments, constitute more than 99% of the organisms. It is anticipated that the number of new QS and QQ systems identified by metagenomic methods will exceed the number identified from single cultures of microorganisms [142,143,144,145]. The usefulness of metagenomic studies is confirmed by many examples in the literature; for example, interesting metagenomic studies on the microbial community, and the distribution of QS and QQ genes in particles of organic material collected from the Yellow Sea. Community structure was shown to be dependent on sampling depth. It was found that luxI and luxR were positively correlated with temperature, while the presence of AHL acylase was positively correlated with depth, SiO_4_^2−^, PO_4_^3−^, and NO_3_^−^, but negatively correlated with temperature and pH [146]. Pyrosequencing of rhizosphere samples from hydroponic potato cultures revealed that the degradation of quorum sensing (AHL) signals in the pathogenic bacterium *Pectobacterium* by *Rhodococcus erythropolis* leads to the stimulation of potato growth [147]. Metagenomic libraries are becoming a valuable tool for the synthesis of new quorum quenching enzymes. One example is the thermostable esterase Est816 from the Tuban basin [148]. On the other hand, the construction of a metagenomic library consisting of several clones from hypersaline soils allowed the identification of the AHL-degrading enzyme with a new family related to the cysteine hydrolase (CHase) group [145]. The analysis of a metagenomic library from pasture soils allowed the identification of a new metallohydrolase with NAHL lactonase activity [149]. A dehydrogenase/reductase (SDR) active in the inactivation of N-(3-oxo-dodecanoyl)-L-homoserine was obtained from a soil metagenome library. The enzyme was found to be effective in reducing the virulence level of Pseudomonas aeruginosa [150]. Based on these literature data, it can be concluded that metagenome research is a very promising prospect and should be continued to obtain new molecules related to QS and QQ.

## 7. Conclusions and Future Perspectives

The prevailing trend in agriculture promotes the development of biological, environmentally friendly methods based on the use of biological control measures. The QS, QSI and QQ enzymes are also part of this trend. The results obtained so far on the effect of QS molecules (essentially AHL) on plants allow us to draw promising conclusions for protecting and increasing the productivity of agricultural crops. The significant effect of AHL-type molecules on plant growth stimulation and resistance to pathogenic organisms is evident. Researchers’ attention is focused on the practical application of QS inhibitors that have the potential to reduce the effectiveness of phytopathogens. Both natural and synthetic inhibitors could be used in practice. Research on the potential use of QS molecules and their inhibitors in agriculture should continue.

## Figures and Tables

**Table 1:** Mode of action of selected natural and synthetic QS inhibitors.

Type of Source Organisms	Inhibitors	Source	QSI Sensitive Microorganism	Effective Concentration of QS Inhibition	Action	Application	References
Higher plants	echinatin	*Glycyrrhiza* L.	*Escherichia coli* O157:H7	50 µM	Inhibition of biofilm formation. Inhibition of EPS ^1^ production. Inhibition of bacterial motility. Reduction in the expression of QS-regulated genes (*luxS*, *pfs*, *lsrB*, *lsrK*, *lsrR*, *flhC*, *flhD*, *fliC*, *csgD*, and *stx2*).	Antimicrobial agents against antibiotic-resistant *E. coli*; potential for treating *E. coli* infection.	[66]
	carvacrol and eugenol	Carvacol from aromatic plants, thyme and oregano. Eugenol from cinnamon and clove oils	*P. carotovorum* subsp. *brasiliense* Pcb1692 *P. aroidearum* PC1	250 μM	Reduction of biofilm formation. Inhibit secretion of PCWDEs ^2^ (i.e., pectate lyase (Pel), polygalacturonase (Peh), and protease (Prt)). Inhibition of AHL production, potentially via direct interaction with ExpI/ExpR proteins. Downregulation of QS-regulated genes (*rsmA*, *acrD* and *nssA*).	Potential for soft rot disease control.	[67]
	phloretin	apple	*Pectobacterium* *brasiliense*	200 µM	Reduction of biofilm formation. Reduction of bacterial motility. Reduction of the secretion of plant cell wall-degrading enzymes. Reduction in AHLs ^3^ production. Inhibition of expi activity. Downregulation of QS-regulated genes (*expI*, *expR*, *luxS*, *rsmB*), plant cell wall-degrading enzymes genes (*pel*, *peh* and *prt*) and motility genes (*motA*, *fim*, *fliA*, *flhC* and *flhD*).	Potential for plant-pathogenic bacteria control.	[68]
Marine organisms	meleagrin	*Penicillum chrysogenium*	*Chromobacterium violaceum*	138.42 µM	Inhibition of bacterial enoyl-acyl carrier protein reductase (FabI).	Antimicrobial agents against antibiotic-resistant human pathogens; potential for treating pathogenic infection.	[69,70]
	alginate oligomer (OligoG CF-5/20)	*Laminaria* *hyperborea*	*Pseudomonas* *aeruginosa*	2%	Inhibition of biofilm formation. Inhibition of bacterial motility. Reduction in AHLs’ production. Alteration in the extracellular production of the pseudomonal virulence factors pyo- cyanin, rhamnolipids, elastase, and total protease. Reduction in the expression of both the *las* and *rhl* systems.	Control chronic infections and biofilm-associated problems of *P. aeruginosa.*	[71,72]
	N-benzyl cinnamamide	*Gracilaria fisheri*	*Vibrio harveyi*	1.66 mg/mL	Inhibition of biofilm formation. RReduction in bioluminescence via inhibition of AI-2 signaling.	Potential antimicrobial drug against *V. harveyi.*	[73]
Bacteria	Amicoumacins	TRM B-02 Taklimakan desert bacterium	*Chromobacterium* *violaceum*	31.25 µg/mL	Inhibition of the violacein biosynthetic pathway via downregulation of the expression of violacein operon A (*vioA*), *vioB*, *vioD* and *vioE* and upregulation of the expression of violacein operon C *vioC*, competitively inhibiting the binding of FAD ^4^ with the *vioD* enzyme.	Antimicrobial agents against antibiotic-resistant human pathogens; potential for treating pathogenic infection.	[74]
	Fatty acyl compounds	*Streptomyces griseoincarnatus* HK12	*Pseudomonas* *aeruginosa* *Staphylococcus aureus*	100 μg/mL	Binding to the conserved sites of substrate binding in the quorum sensing system, LasI.	Antimicrobial agents against crucial nosocomial respiratory pathogen.	[75]
	Cyclic dipeptides (CDPs)	*Pseudomonas aeruginosa* RKC	*Lelliottia amnigena* RCE	10 mg/mL	Regulation diverse metabolites of the pathogen diketopiperazine. Inhibition of QS-mediated pathogenicity via competitive binding with receptors	Potential for soft rot disease control.	[76]
Synthetic compounds	synthetic peptides (LIVRHK and LIVRRK)		*Pseudomonas* *aeruginosa* PAO1	100 μg/mL	Inhibition of biofilm formation. Inhibits the production of virulence factors, including pyocyanin, protease, and rhamnolipids. downregulation of the expression of genes *lasI*, *lasR*, *rhlI*, and *rhlR.*	Control chronic infections and biofilm-associated problems of *P. aeruginosa.*	[77]
	N-acyl-2-aminopyrimidine derivatives		*Acinetobacter* *baumannii*	3.8 μM	Inhibition of biofilm formation. Reduction in EPS production. Reduction of bacterial motility.	Antimicrobial agents against antibiotic-resistant human pathogens; potential for treating pathogenic infection.	[78]
	PQIs (phc quorum sensing inhibitors)		*Ralstonia solanacearum* OE1-1	41.2 nM– 731 μM depends on (R)- or (S)- enantiomers	Act as competitive antagonists of 3-OH MAME ^5^. Inhibition of QS-dependent gene expression; repression inhibition of the production of ralfuranone and EPS.	Potential for plant-pathogenic bacteria control.	[79]

^1^ EPS—extracellular polysaccharide; ^2^ PCWDEs—plant cell wall-degrading enzymes; ^3^ AHLs—N-acyl-homoserine lactones; ^4^ FAD—flavin adenine dinucleotide; ^5^ 3-OH MAME—(R)-methyl 3-hydroxymyristate.

**Table 2:** The effects of the interactions of AHL molecules with different carbon chain lengths.

Autoinducer	Plant	Impact Effects	References
C6-HSL 3O-C6-HSL 3O-C8-HSL	*Arabidopsis thaliana*	Main root growth stimulation.	[115,116,117,118,119]
3O-C10-HSL	*Vigna radiata*	Adventitious roots growth stimulation.	[17]
C4-HSL C6-HSL 3OHC4-HSL 3OHC6-HSL	*Phaseolus* L. *Solanum lycopersicum*	Systemic resilience similar to ISR.	[120]
C10-HSL	*Arabidopsis thaliana*	Inhibition of main root growth. Adventitious roots growth stimulation. Root hairs growth stimulation.	[121]
3O-C8-HSL 3O-C14-HSL	*Arabidopsis thaliana*	Increased resistance to *Pseudomonas syringae pv. tomato* DC3000.	[16,18]
3O-C14-HSL	*Medicago truncatula*	Increased root nodulation.	[122]
C4-HSL C6-HSL	*Solanum lycopersicum*	Increasing the content of salicylic acid. PR1a induction. Chitinase induction.	[123]
C6-HSL C8-HSL	*Arabidopsis thaliana*	Root growth stimulation by GCR1/GPA1 genes.	[117]
3-okso-C14-HSL	*Arabidopsis thaliana*	Stimulating the expression of antioxidant and defense genes through the oxylipin and salicylic acid pathway.	[124]
C8-HSL C10-HSL	*Hordeum* L. *Triticum* L.	Root stimulation. Increasing the production of phase II antioxidant and detoxifying enzymes.	[125,126]
